# Fatal Self-Inflicted Chop Wound by a Supari Cutter (Sudi): A Rare Case With Forensic Analysis of Wound Patterns and Weapon Biomechanics

**DOI:** 10.7759/cureus.114344

**Published:** 2026-08-11

**Authors:** Vimal Kanna G, Archit M Modi

**Affiliations:** 1 Forensic Medicine and Toxicology, Sumandeep Vidyapeeth Deemed to be University, Vadodara, IND; 2 Medicine, Sumandeep Vidyapeeth Deemed to be University, Vadodara, IND

**Keywords:** cervical injuries, chop wound, financial problems, manner of death, neck injuries, self-inflicted wound, sudi, suicide, suicide mimicking homicide, weapon biomechanics

## Abstract

Chop wounds on the body, produced by the forceful impact of a heavy swinging blade, are conventionally considered telltale signs of homicide in forensic pathology, as a person cannot plausibly inflict such a wound upon themselves, since it appears to demand the momentum and arc of another individual's swinging arm. Existing literature has extensively documented suicidal deaths involving incised and stab wounds, where the mechanics of a short blade wielded by the victim's own hand are well established and readily accepted. Until now, however, no prior study has documented such a case of suicide resulting from a chop wound.

The case describes a rare instance in which a 51-year-old man died from a self-inflicted chop wound to his own neck. He did not employ a conventional blade but instead used a Sudi, a device commonly found in Indian shops and ordinarily used for cutting areca nut or betel nut. He positioned his neck within the cutting mechanism of the Sudi and self-actuated its lever, producing a deep, transecting wound that divided the cervical soft tissues, the major neurovascular structures, the cervical vertebra, and the spinal cord. What renders this case forensically noteworthy is that the morphology of the wound did not conform to the pattern typically associated with an assault delivered by a swinging blade. Instead, forensic examination identified asymmetric wound depth, bevelling along the wound margins, and pressure and traction marks on the adjacent skin. These features could not be reconciled with the conventional arc and impact mechanics of a wielded chopping weapon. They became fully explicable once the compressive and shearing action of the Sudi's lever-based mechanism was taken into account.

The Sudi does not operate through a freely swung strike but rather functions through a hinged lever that compresses and shears through tissue placed within its jaws. Consequently, the wound superficially resembled a homicidal chop injury while actually bearing the mechanical signature of a self-actuated, static compression event. It must be emphasized that no single category of evidence was sufficient in isolation. Rather, it was the convergence of multiple strands like the findings at the crime scene, the contextual history of the deceased's financial distress and a rigorous biomechanical analysis of the weapon that proved pivotal in establishing suicide as the definitive manner of death. This case demonstrates concretely how the reflexive assumption that chop wounds are inherently homicidal can mislead investigators if the specific mechanics of the implicated weapon are not meticulously scrutinized.

Exceptions can and do arise when atypical weapons with unconventional mechanical actions are involved. By documenting morphological features that diverged from the expected homicidal pattern and by tracing those divergences to the specific engineering of the Sudi's cutting action, the report illustrates how the boundary between homicide and suicide can sometimes become obscured. Features characteristic of one manner of death may, paradoxically manifest in a case belonging to the other. Forensic pathologists and investigating authorities must resist the temptation to arrive at hasty conclusions regarding manner of death based on wound appearance alone. A comprehensive and evidence integrated approach is essential, one in which weapon biomechanics, scene reconstruction, and circumstantial history are weighed collectively.

## Introduction

Suicide is a leading cause of preventable death worldwide, claiming over 727,000 lives annually by conservative estimates, with a substantial burden of underreporting [1]. India carries a disproportionate share of this toll contributing more than a quarter of global suicidal deaths, with hanging, poisoning, self-immolation, and drowning constituting the predominant methods [2-4]. Sharp force, by contrast, accounts for a minority of suicidal deaths globally, with published estimates placing its prevalence between 1.6% and 3% [5]. When sharp force suicide does occur, it usually follows a recognisable pattern. Incised wounds inflicted by light implements such as razor blades or knives and are concentrated at anatomically accessible sites like the wrists, the antecubital fossa and the anterior neck, consistent with the reach and dominance of the victim's own hand [5] . Tentative or "hesitation" marks, representing abortive early attempts before the final act, are present in more than three-quarters of cases and constitute one of the most reliable indicators of suicidal intent [6]. 

Chop wounds occupy an entirely different forensic category. Produced by heavy, sharp-edged instruments like axes, cleavers, swords, they are delivered with significant swinging momentum causing deep tissue-cleaving injuries that might transect bone and cartilage as well, causing near-instantaneous incapacitation [7]. For these reasons, chop wounds are widely regarded in forensic practice as the hallmark of homicidal assault as the physical force required to deliver such a blow has traditionally been considered beyond the capacity of self-infliction [7-9]. Suicidal chop wounds represent an almost uncharted entity in the forensic literature and their occurrence presents a serious diagnostic challenge to the forensic pathologist.

We report a case of fatal self-inflicted chop wound to the neck, by means of a traditional betel nut cutter known as an Sudi - a hinged, lever-operated blade device widely used in betel nut (supari) shops across rural India. Unlike a conventional chopping weapon whose destructive energy derives from gravitational momentum and muscular swing, the Sudi operates through a fixed fulcrum: the blade descends along a defined arc when the lever arm is depressed. This mechanism allows a victim to position their neck beneath the blade and self-actuate the cut with minimal upper limb force, producing a deep, transecting wound that superficially resembles a homicidal chop but carries a fundamentally distinct biomechanical signature. We present the autopsy findings, analyse the wound morphology in the context of the device's mechanics, and discuss the forensic implications of this unusual case.

## Case presentation

**Figure 1:**
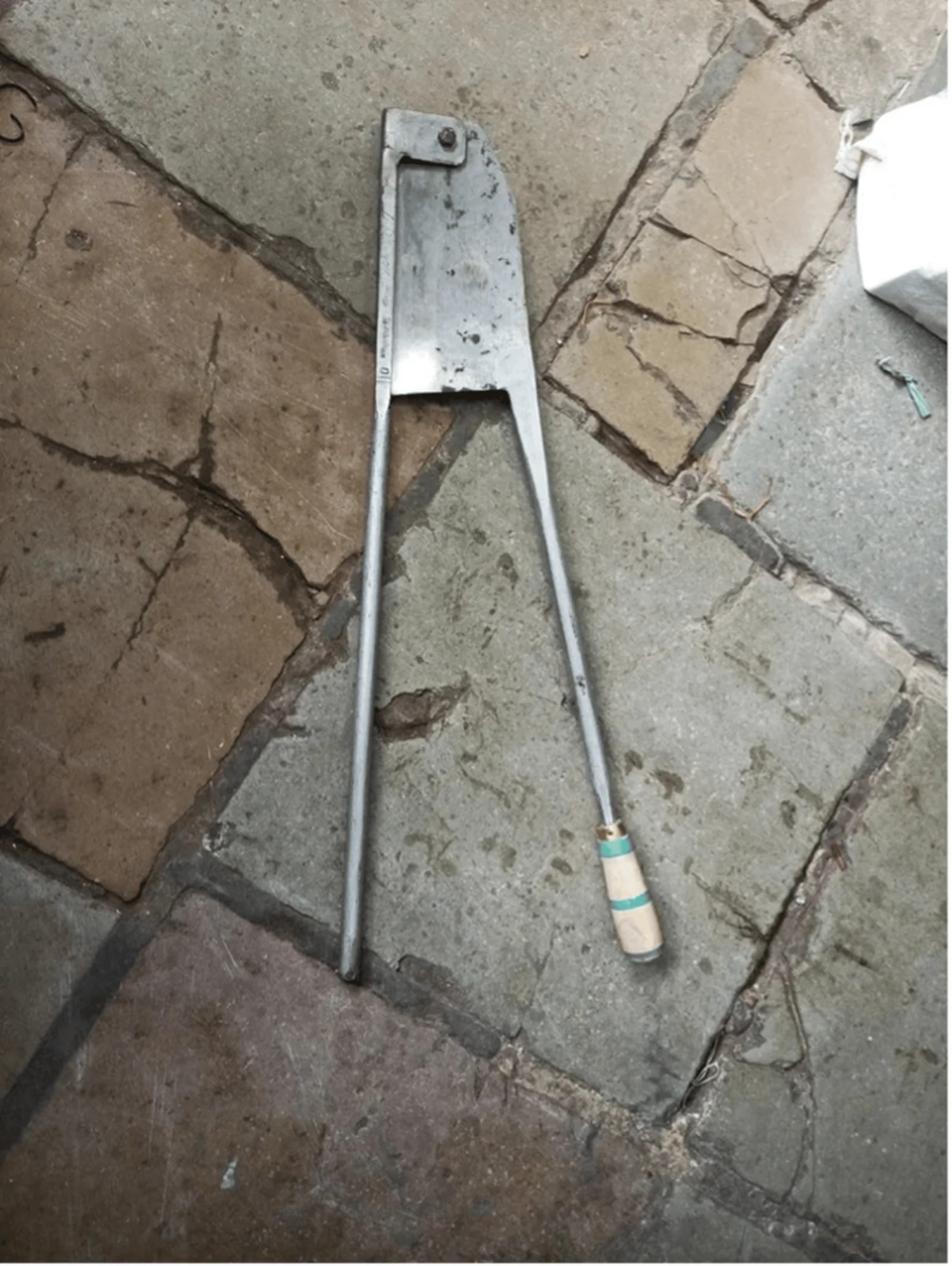
An illustrative image of the Sudi: a conventional tobacco-cutting tool prevalent across India

A 51-year-old male businessman reportedly experiencing considerable financial hardship visited the shop of an acquaintance who sold betel nut (supari). The shop housed a traditional Sudi, a hinged lever-operated tobacco cutter (Figure 1).

**Figure 2:**
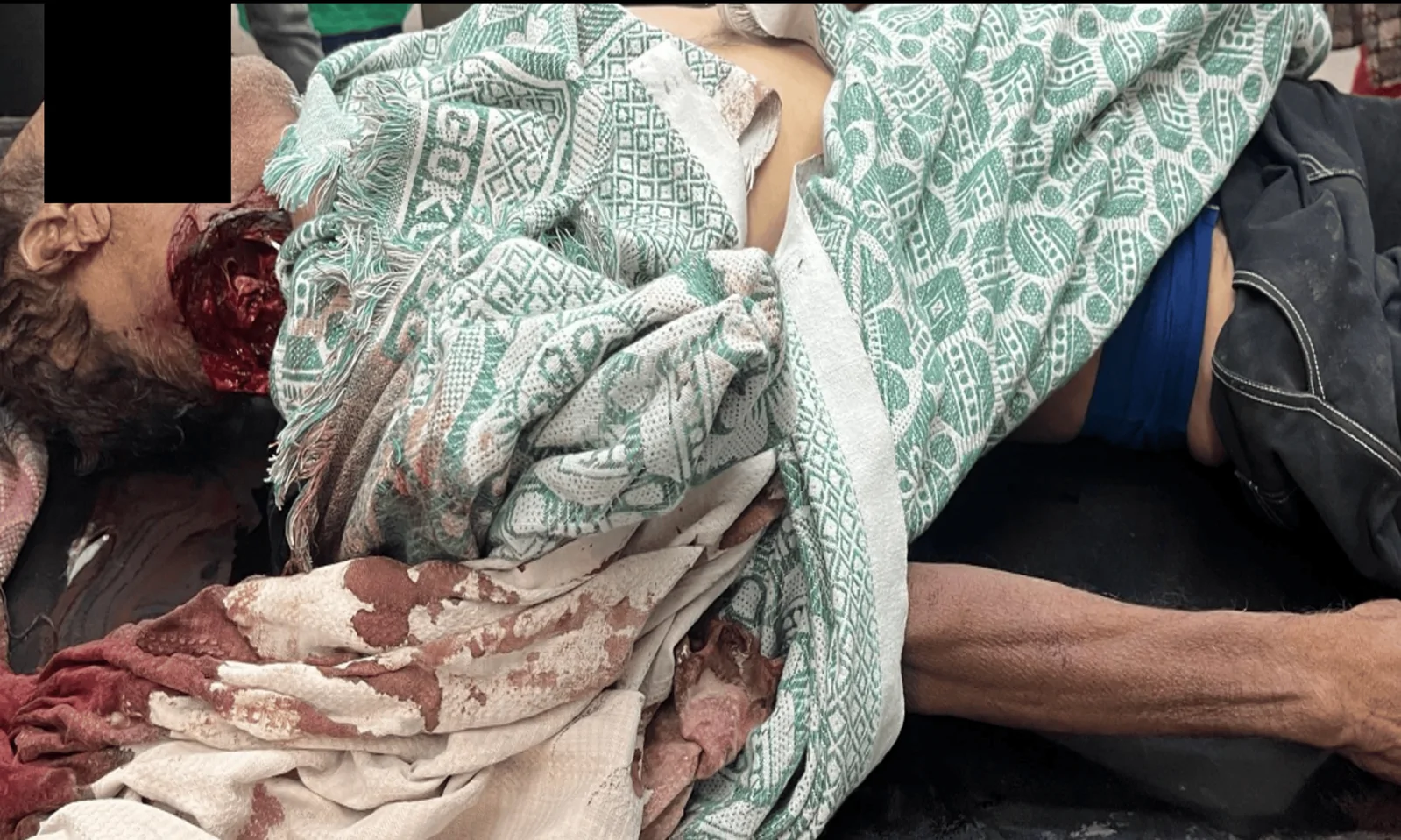
The body of the deceased victim

At approximately 6:00 p.m., the deceased was found unconscious on the floor of the shop in a pool of blood. He had sustained a deep wound to the right side of the neck. Emergency services were contacted, and the patient was transported to a nearby hospital where resuscitation and life-sustaining interventions were instituted. Despite these efforts, the patient could not be revived and was declared dead at approximately 7:00 p.m. The body was subsequently referred for post-mortem examination (Figure 2).

Autopsy findings

**Figure 3:**
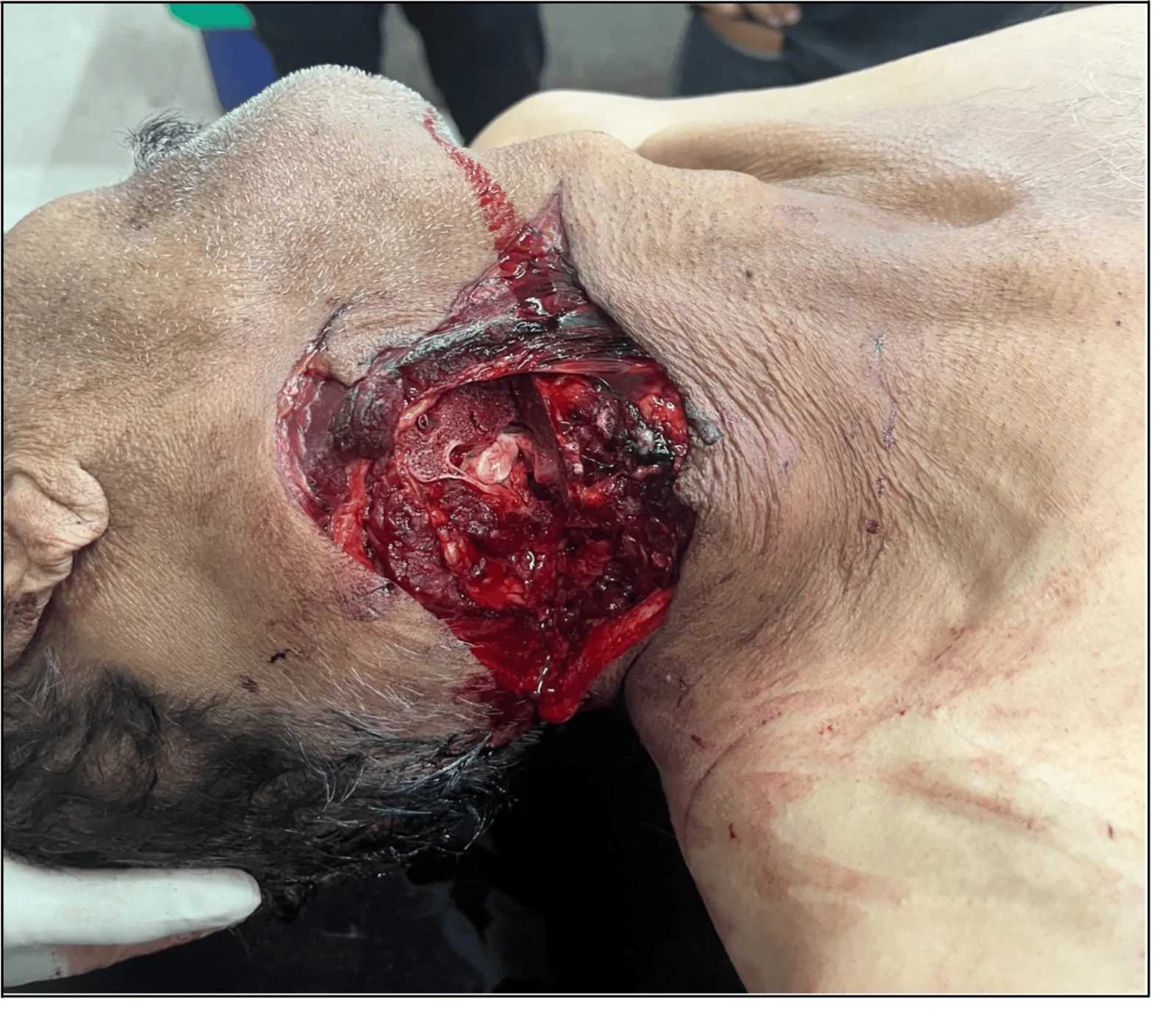
The open wound on the right side of the neck

External examination revealed a single, well-demarcated chop wound over the right side of the neck, measuring 15 cm in length and 6 cm in width, positioned 2 cm to the right of the midline and extending posteriorly till the nape of neck (Figure 3).

The wound margins were sharp and showed adherent clotted blood. The overall orientation of the wound was consistent with the trajectory of the Sudi's blade arc.

**Figure 4:**
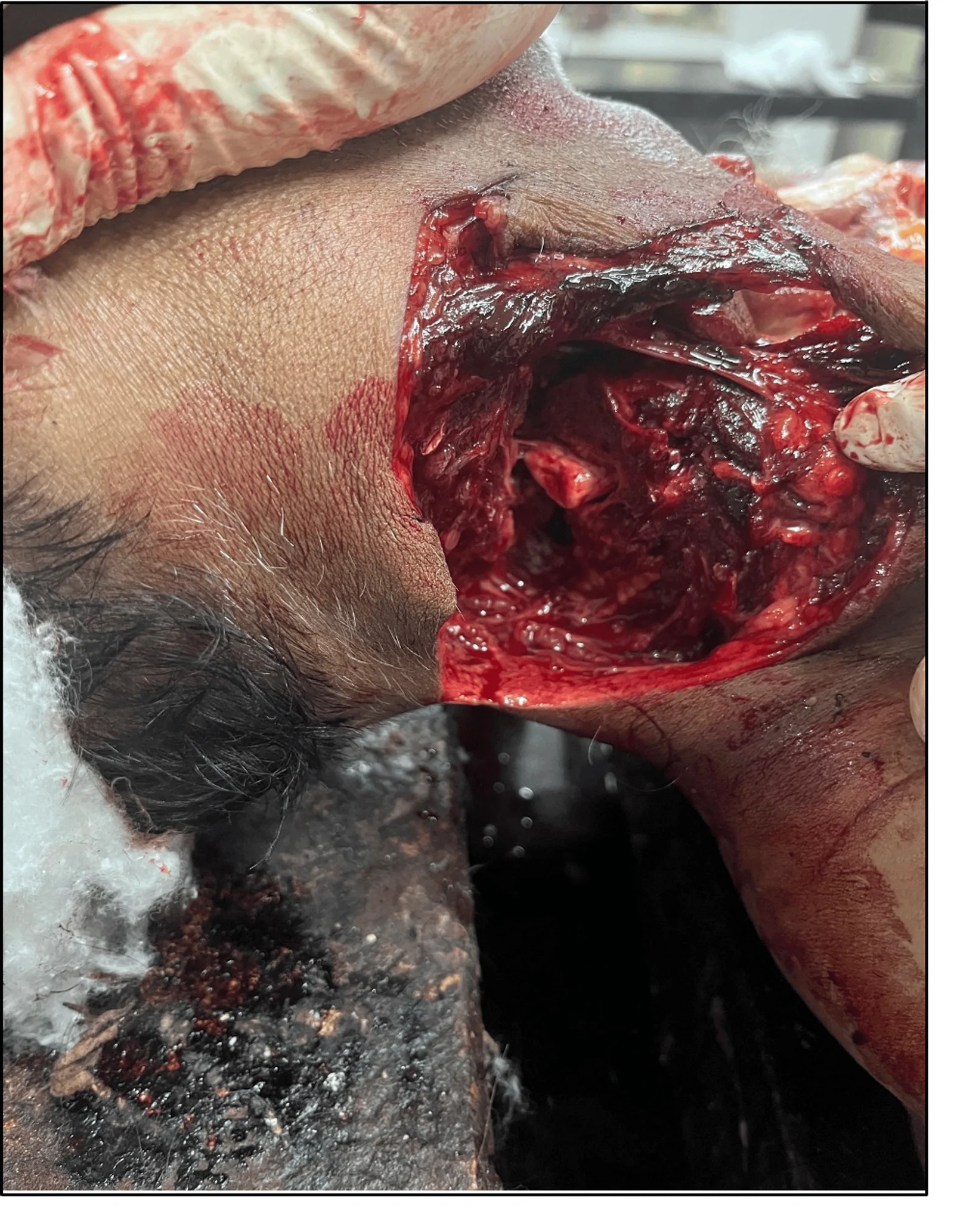
The wound revealing significant damage to internal structures, consistent with the causative weapon

On further exploration, the wound displayed notably asymmetric depth, significantly deeper on the right side, directly corresponding to the point of initial blade contact at the fulcrum end of the lever. The underlying structures were sharply transected at a single level, including the cervical musculature, the right-sided neurovascular bundle (major blood vessels and nerves of the neck), the cervical vertebra, and the spinal cord. Crucially, the skin margins exhibited bevelling and distinct friction and traction marks, consistent with a compressive shearing force rather than the clean, high-velocity cut produced by a swinging blow (Figure 4).

The absence of "tailing", the tapering linear extension typically seen when a blade is drawn across skin in a slicing motion, further corroborated that the mechanism was direct vertical pressure via the lever, not a swinging or dragging action. No hesitation marks, defensive injuries or additional wounds were identified on the body. The Sudi device, recovered from the scene with blood present on the blade, demonstrated a blade arc and fixed hinge geometry consistent with the wound's orientation, depth asymmetry, and position relative to the midline.

## Discussion

To our knowledge, this is the first reported case of a fatal, self-inflicted chop wound to the neck using Sudi in the forensic literature. Its significance lies not merely in its rarity, but in what it reveals about the limits of conventional wound classification when applied without contextual analysis. 

Forensic classification of sharp force injuries

Sharp force injuries are conventionally divided into three types: incised wounds (produced by a drawing or slicing motion), stab wounds (produced by thrust), and chop wounds (produced by the momentum of a heavy, swinging edge). This taxonomy has clear forensic utility in the vast majority of cases, but it carries an implicit assumption that the wounding mechanism can be inferred from the wound's gross morphology alone. The distinction between suicidal and homicidal sharp force injuries has been examined in several retrospective studies, which have identified reliable, though not infallible, predictors for each manner of death. Wounds at the neck and upper limbs, the presence of hesitation marks, and isolated cut wounds are consistently associated with suicide; injuries to the head, back, or hands, the presence of defensive wounds, and clothing damage tend toward homicide [10,11].

In a retrospective analysis of 118 sharp force fatalities over 22 years, Brunel et al. [7] found that bone or cartilage involvement was a statistically significant predictor of homicide, a finding that underscores why the present case, with its vertebral transection, would prima facie invite a homicidal interpretation. 

Chop wounds occupy a firmly homicidal position in classical forensic teaching. The physical requirements of a conventional chop - a heavy implement, a muscular swing, sustained momentum - have historically placed self-infliction beyond credible consideration. Karger et al. [6], in a landmark series of 65 sharp force suicides spanning nearly three decades, documented that deviation from typical suicidal patterns occasionally occurs, and attributed these "bizarre" atypical cases to either unusual weapons or extraordinary determination on the part of the victim. The present case fits precisely within this framework: the Sudi is an unusual weapon, in both conventional forensic and biomechanical terms, and its mechanism effectively circumvents the physical barrier that ordinarily renders self-inflicted chop wounds implausible. 

Sudi as a mechanical enabler

The critical distinction between the Sudi and a conventional chopping instrument lies in the source and delivery of wounding force. A swinging axe or sword derives its energy from muscular momentum applied over a distance where the wounding force scales with the mass of the instrument and the velocity of the swing as it requires the full kinetic engagement of the attacker's upper limbs and core. The Sudi, by contrast, is a fixed-fulcrum lever system: the blade travels along a predetermined arc, and the force required to actuate it is a function of lever mechanics, not muscular swing. A person placing their neck at the blade's resting position and depressing the handle arm can deliver a wound of considerable depth with comparatively little effort and can do so without requiring an accomplice, a swing, or the spatial freedom of movement that a conventional chopping assault demands. 

This mechanical principle, the use of an environmental tool or device to overcome the physical barriers to self-infliction, has precedent in the forensic literature. Janík et al. [10] reported a case of suicide in which a 79-year-old man used a self-modified circular table saw to inflict multiple saw-type impacts to the head and neck, resulting in partial decapitation. Like the Sudi, the circular saw converted a mechanically demanding injury into one achievable through self-actuation of a fixed device. That case similarly challenged initial assumptions of homicide and required detailed technical examination of the instrument before the manner of death could be established. Sartori et al. [11], in a 2025 series of three cases in which suicide was initially mistaken for homicide due to atypical sharp force patterns, concluded that a multidisciplinary approach incorporating autopsy findings, scene investigation, and psychosocial history is essential whenever the injury morphology defies standard classification.

Wound morphology as the key differentiating evidence

The wound profile in this case is morphologically distinguishable from a homicidal chop wound on several grounds. A homicidal chop, delivered by a swinging weapon, typically produces a wound of relatively uniform depth along its long axis, or a "boat-shaped" defect where the deepest point corresponds to the apex of the blade's arc and depth tapers symmetrically toward both ends. In contrast, the wound in this case showed pronounced asymmetry, substantially deeper on the right side, corresponding precisely to the point of handle-side blade contact. This uneven depth is biomechanically inconsistent with a swinging blow but entirely consistent with the progressive compression of a lever-actuated blade descending from a fixed pivot point. 

Additionally, the presence of bevelling and distinct friction and traction marks along the wound margins indicates a pressure-traction mechanism, where skin is simultaneously compressed and drawn into the wound track as the blade advances. This pattern is distinct from the clean, momentum-driven incision of a swinging weapon, which typically produces sharply defined, non-bevelled margins. The absence of "tailing", the tapering linear extensions typically seen when a blade is drawn laterally across skin in a slicing motion, further corroborated that the blade's trajectory was directly vertical rather than sweeping. Taken together, these morphological features constituted a recognisable signature of the Sudi's lever mechanics, and allowed a confident distinction from conventional chopping assault. 

The distinction between wound morphology produced by different sharp-force mechanisms underscores the observation of Al Gheryafi et al. [8] in their scoping review: that no single parameter reliably establishes manner of death in sharp force fatalities, and that integrated forensic evaluation combining wound analysis, scene reconstruction, weapon examination, and circumstantial context is essential. This principle is further supported by Terranova et al. [12], who found that in atypical sharp force fatalities, psychosocial and circumstantial context, specifically the identification of plausible explanatory motives, significantly strengthened the determination of suicide. 

Forensic and investigative implications

This case identified a clear potential for misclassification when wound morphology is assessed in isolation. A large, deep chop wound to the neck, transecting vertebral bone, in the apparent absence of hesitation marks, would by pattern-matching alone strongly suggest homicide. The absence of tentative injuries here does not undermine a suicidal conclusion; Karger et al. [6] noted that approximately a quarter of suicidal sharp force cases present without hesitation marks, particularly when the victim employs a device or mechanism that does not allow for an incremental approach. Sartori et al. [11] likewise documented suicidal cases without hesitation wounds, where the method itself precluded tentative attempts. 

The scene evidence was central to establishing the correct manner of death: the Sudi was present with blood on the blade, the body position was consistent with the device's geometry, and no evidence of a third party's presence was identified. The deceased's financial distress, while not a diagnostic criterion, provided contextual plausibility for suicidal intent. 

For the forensic pathologist, this case illustrates the danger of what might be called mechanism-based anchoring, the premature assignment of manner of death based on the perceived requirements of the wounding mechanism, without fully interrogating the specific instrument involved. Traditional forensic teaching appropriately associates chop wounds with homicide, because the assumption that self-infliction is mechanically impracticable is valid for the vast majority of heavy swinging weapons. It fails, however, when the instrument operates on a fundamentally different mechanical principle. When atypical injuries are encountered, a detailed analysis of every available instrument, its geometry, fulcrum mechanics, required actuation force and wound-producing trajectory must precede any conclusion about manner of death. 

## Conclusions

We present the first documented case of a fatal, self-inflicted chop wound to the neck, inflicted using the lever-actuated blade mechanism of a traditional tobacco cutter (Sudi). The wound's morphological features, including asymmetric depth, bevelling, and pressure-traction marks, were not consistent with a conventional swinging chop but were fully explicable by the biomechanics of the device. This case demonstrates that unconventional instruments can produce injuries that superficially mimic homicidal patterns, and thereby underscores the critical importance of integrating weapon analysis and scene reconstruction into forensic wound interpretation. Classification of manner of death must be grounded in complete, contextual evidence. The reliance on conventional wound taxonomy alone, without interrogating the specific mechanics of the instrument involved, risks a fundamental misattribution that could carry serious medicolegal consequences.
